# Whole-cycle analysis of echocardiographic tissue Doppler velocities as a marker of biological age

**DOI:** 10.3389/fcvm.2022.1040647

**Published:** 2023-01-04

**Authors:** Joanna Nan Wang, Niels Thue Olsen, Ida Arentz Taraldsen, Rasmus Mogelvang

**Affiliations:** ^1^Department of Cardiology, The Heart Centre, Copenhagen University Hospital – Rigshospitalet, Copenhagen, Denmark; ^2^Copenhagen City Heart Study, Copenhagen University Hospital – Bispebjerg and Frederiksberg Hospital, Frederiksberg, Denmark; ^3^Department of Cardiology, Copenhagen University Hospital – Herlev and Gentofte, Copenhagen, Denmark; ^4^Faculty of Health and Medical Sciences, Institute of Clinical Medicine, University of Copenhagen, Copenhagen, Denmark; ^5^Cardiovascular Research Unit, University of Southern Denmark, Svendborg, Denmark

**Keywords:** tissue Doppler imaging, healthy aging, cardiac degeneration, accelerated aging, biological age

## Abstract

**Purpose:**

Tissue Doppler imaging (TDI) is a sensitive marker of impaired cardiac function and different phases of the TDI curve carry different prognostic information. It is not known how continuous TDI curves change with age in normal subjects, and whether these changes differ from changes seen in individuals at risk of future cardiac events.

**Methods:**

A total of 1,763 individuals from the general population were examined with color TDI at the septal and lateral mitral sites. A low-risk group was defined as without cardiac risk factors (hypertension, diabetes or ischemic heart disease) at baseline and without any cardiac events (cardiovascular death or admission due to either heart failure or acute myocardial infarction) during 10-years follow-up. All TDI curves were corrected for heart rate, and whole-cycle analysis of age-related changes to TDI velocities was performed in both low-risk (*n* = 881) and high-risk individuals (*n* = 882).

**Results:**

In the low-risk population, four phases where myocardial velocity differed most (*p* < 10^–10^) according to age were identified [in a standardized cardiac cycle of 1 second (s)]: Systolic peak (0.09–0.13 s), systolic plateau (0.18–0.27 s), early diastole (0.43–0.54 s) and late diastole (0.88–0.95 s). With increasing age, systolic velocities decreased, early diastolic velocities decreased and had delayed peak, and late diastolic velocities increased until age 70 and then stopped increasing. In the high-risk population, comparison to corresponding age groups of the low-risk population showed: Lower early diastolic velocities in 20–40-year-olds; higher late diastolic velocities and lower peak systolic velocities in 40–60-year-olds; further decreased systolic velocities including the systolic plateau and decreased late diastolic velocities in 60-year-olds. The time segments around the systolic peak (*p* = 0.002) and early diastole (*p* < 0.001) differed significantly between the high-risk and low-risk population, thus making it possible to use the individual age gap between a TDI-derived biological age and the real chronological age as a tool to discriminate high-risk individuals from low-risk individuals.

**Conclusion:**

We found that individuals with cardiac risk factors display findings compatible with an accelerated aging of the heart and thus propose TDI-derived biological age as a tool to identify high-risk patients.

## Introduction

Measurement of myocardial velocities with tissue Doppler imaging (TDI) has become an integrated part of the assessment of diastolic heart function in clinical echocardiography (1). Both systolic as well as diastolic TDI velocities have been demonstrated to be sensitive markers of impaired cardiac function (2–4) and prognosis (5–7), and there is a significant association between TDI peak values and age (8–10). While current recommendations and existing studies mainly focus on peak values of TDI velocities, it has been shown that different phases of the TDI curve carry different prognostic information (11). It is not known how continuous TDI curves change with increasing age in normal subjects, and whether these changes differ qualitatively from changes seen in individuals at risk of future adverse events. Computer-aided automated analysis of TDI curves could allow the integration of information from a large number of examinations to answer these questions.

Thus, we set out to achieve the following aims: (1) Perform whole-cycle analysis of TDI myocardial velocities in a low-risk population without cardiac risk factors to determine age-related changes. (2) Compare whole-cycle TDI velocities in high-risk individuals with cardiac risk factors to low-risk individuals at different age levels. (3) Relate the effect of cardiac risk factors on TDI velocities with changes during normal aging.

## Materials and methods

### Study population

This study is an echocardiographic sub-study of the 4th Copenhagen City Heart Study, a Danish cohort study of cardiovascular disease and risk factors (12, 13). The present study includes 1,763 randomly selected men and women (20–93 years old) from the general population who underwent an echocardiographic examination including color TDI. Whether a participant underwent echocardiography was independent of health status and other risk factors. Individuals with atrial fibrillation or significant valvular stenosis or regurgitation were excluded. All subjects gave informed consent to participate. The study was performed in accordance with the Helsinki Declaration and approved by the regional ethics committee.

### Health examination

Systolic and diastolic blood pressures were measured on the left upper arm, in a sitting position after 5 min of rest. Hypertension was defined as systolic blood pressure ≥ 140 mmHg, diastolic blood pressure ≥ 90 mmHg or use of antihypertensive medication. Diabetes was defined as plasma glucose concentration ≥ 11.1 mmol/L, HbA_1c_ level > 7.0%, self-reported disease or use of insulin or another antidiabetic medication. Ischemic heart disease (IHD) was defined as ischemic alterations on the electrocardiogram (Minnesota codes 1.1–3) or a history of hospital admission due to acute coronary artery occlusion, percutaneous coronary intervention or coronary artery bypass grafting.

### Tissue Doppler imaging

Three experienced echo technicians performed all echocardiograms. Color TDI loops were obtained in the apical four-chamber view at the highest possible frame rate, using GE Vingmed Ultrasound’s Vivid Five with a 2.5 MHz probe (Horten, Norway). For each individual, one TDI loop and the corresponding electrocardiogram were saved and analyzed offline by investigators blinded to other information. TDI velocity curves were obtained by measuring within a 6 mm circular region of interest at the septal and lateral mitral annular positions. The data of the septal and lateral TDI velocity curves of one heart cycle and the concurrent electrocardiogram were saved in one CSV file for each individual.

### Outcome and definitions

Participants were followed from the examination in 2001 through 2003 until April 2013 or time of event. The primary endpoint was a cardiac event within 10 years of follow-up, defined as the combined endpoint of cardiovascular death or admission due to either heart failure or acute myocardial infarction. Low-risk individuals were defined as being free of hypertension, diabetes and IHD at baseline and without any cardiac event at 10 years of follow-up. High-risk individuals were defined as having one or more of the following: Hypertension, diabetes or IHD at baseline, or cardiac event within 10 years of follow-up. Follow-up data on cardiovascular deaths were collected from the national Danish Causes of Death Registry. Follow-up data on admissions with heart failure and acute myocardial infarction were obtained from the Danish National Board of Health’s National Patient Registry. Follow-up was 100% complete.

### Data analysis

#### Importing data

In order to analyze the entire TDI curve of all individuals at once, all the CSV files were imported into a 3D array. Each 2D layer in the array was labeled with a unique study-ID and contained the values of the TDI curves and electrocardiogram of that individual. Baseline data and follow-up data were saved in separate data frames but labeled by the same unique ID, making all data easily accessible for analysis.

#### Standardizing heart cycles

In order to standardize the length of the TDI curves of individuals with different heart rate, heart cycles were standardized to a length of 1 s in the following way:

- **Defining starting point of heart cycle:** Time of R-peak in the electrocardiogram was defined as the starting point of time = 0 in the standardized heart cycle.
- **Identifying length of systole and diastole:** Start of systole was defined as time = 0. End of systole was defined as the time of the first negative TDI value (either septal or lateral) after 0.2 s. The limit of 0.2 s was set to avoid the initial negative TDI values at the beginning of systole (isovolumetric contraction). Diastole was defined as the rest of the heart cycle from end of systole.
- **Standardizing length of systole:** Fridericia’s cube-root formula for QTc correction was used to correct the duration of systole to fit a heart cycle of 1 s (14). The values of the formula *QTc* = *QT/RR^1/3^* were: QT = length of systole; RR = length of heart cycle = maximum time value of the TDI curve. Application of this method meant that all the systolic time values of the TDI curve were divided by RR^1/3^.
- **Standardizing length of diastole:** First, the time values of the diastole were parallel shifted to fit the new time position of the end of systole. Afterward, all the time values were equally distributed between the time of end of systole to time = 1 (end of heart cycle).
- **Standardizing amount and frequency of time observations:** Due to different cycle lengths and the standardization process, different individuals now had a different amount of time observations and time values. To make further analyses possible, we defined that all individuals should have TDI values each 0.01 s, making up 101 observations from *t* = 0.00 to *t* = 1.00. To impute TDI values at the exact 0.01 time-intervals, we made a linear fit through the TDI values of the two nearest available time data points to derive the TDI value that fitted the newly created 0.01 time-position.

#### Regression-based TDI velocities

**FIGURE 1:**
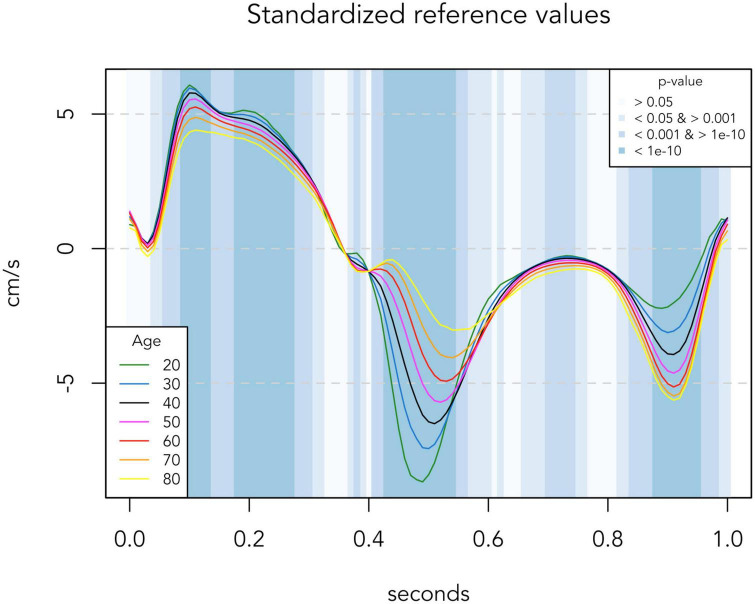
Regression-based reference TDI curves of the low-risk population (*n* = 881). Colored lines represent reference TDI curves for each age. Background colors indicate significance levels of the association between age and TDI values at each 0.01 s time-position. TDI values are averaged for septal and lateral mitral sites, presented for one standardized heart cycle of 1 s.

To generate reference TDI velocities for any given age, we made a regression model based on all the TDI curves of the low-risk population. Septal and lateral TDI values for each individual were averaged before performing regression. Linear regression with a quadratic age-variable was performed in all 101 time-positions (from *t* = 0.00 to *t* = 1.00). A quadratic age-variable was chosen because of non-linearity in some time-positions. Normality was checked with QQ-plots. In Figure 1, the presented normal reference TDI velocities are generated from this regression model, and *p*-values were calculated for each 0.01 time-position. In Figure 3, regression curves for the high-risk population were generated from a similar regression model based on the data of the high-risk population. The *p*-values of the age-adjusted association between TDI values and risk group were calculated for each 0.01 time-position.

#### Biological age

Based on the data of the low-risk population, a regression model was made for the association between age and the mean TDI values of each of the two important phases of the heart cycle identified in Figure 3 (systolic peak and early diastole), respectively. Then, for each individual, a biological age was calculated for each phase by inserting their mean TDI values of the phase into the corresponding regression model. Then we subtracted their real chronological age from their calculated biological age, yielding an individual age gap. These individual age gaps are presented in Table 3 for each cardiac risk group, including an average age gap based on both phases.

#### Statistics

In Table 1, Welch two-sample *t*-test was used for continuous variables and Chi-square test for categorical variables. In Table 2, comparisons between groups were done by ANOVA. In Table 3, Welch two-sample *t*-test was used to compute *p*-values in comparison to the reference (low-risk) group of same TDI area.

**TABLE 1:** Population characteristics.

	Low-risk	High-risk	*p*-value
***n***	**881**	**882**	
Male sex, *n* (%)	372 (42)	389 (44)	0.45
Age, years (SD)	49.2 (±14.6)	66.3 (±12.0)	<0.001
BMI, kg/m^2^ (SD)	24.4 (±3.3)	26.4 (±4.1)	<0.001
Heart rate, bpm (SD)	65 (±10)	69 (±11)	<0.001
Hypertension, *n* (%)	0	742 (84)	
Diabetes, *n* (%)	0	179 (20)	
IHD, *n* (%)	0	231 (26)	
BBB, *n* (%)	11 (1.3)	36 (4.1)	<0.001
LVEF,% (SD)	59.9 (±1.0)	59.6 (±2.5)	0.001
LAVI, mL/m2 (SD)	18.1 (±5.2)	20.0 (±7.0)	<0.001
E,m/s (SD)	0.75 (±0.16)	0.68 (±0.16)	<0.001
A,m/s (SD)	0.62 (±0.15)	0.77 (±0.18)	<0.001
DT, ms (SD)	160 (±40)	180 (±40)	<0.001
Creatinine, μmol/L (SD)	79.3 (±13.8)	81.6 (±18.8)	0.003
Cholesterol, mmol/L (SD)	5.3 (±1.1)	5.8 (±1.1)	<0.001
Cardiac event at 10-year follow-up, *n* (%)	0	202 (23)	
- AMI admission, *n* (%)	0	56 (6.3)	
- HF admission, *n* (%)	0	101 (11.5)	
- Cardiac death, *n* (%)	0	107 (12.1)	

Baseline characteristics and follow-up data. High-risk group defined as the presence of hypertension, diabetes or ischemic heart disease at baseline or the occurrence of a cardiac event at 10 years of follow-up. Continuous variables presented as mean (± SD). Categorical variables presented as *n* (%). BMI, body mass index; bpm, beats per minute; IHD, ischemic heart disease; BBB, bundle branch block; LVEF, left ventricle ejection fraction; LAVI, left atrial volume index; E, transmitral E-wave; A, transmitral A-wave; DT, deceleration time; AMI, acute myocardial infarction; HF, heart failure.

**TABLE 2:** Mean peak velocities by age group.

	Low-risk				High-risk			
	**20–40 years**	**40–60 years**	**> 60 years**	***p***	**20–40 years**	**40–60 years**	**>60 years**	***p***
s’ (cm/s)	6.7 (± 1.3)	6.2 (± 1.3)	5.7 (± 1.5)	*p* < 0.001	6.9 (± 1.4)	5.8 (± 1.2)	5.2 (± 1.2)	*p* < 0.001
e’ (cm/s)	10.8 (± 2.0)	8.5 (± 1.9)	6.2 (± 1.8)	*p* < 0.001	9.7 (± 1.7)	7.0 (± 1.9)	5.1 (± 1.6)	*p* < 0.001
a’ (cm/s)	4.4 (± 1.5)	6.2 (± 1.7)	7.4 (± 1.8)	*p* < 0.001	5.6 (± 1.9)	6.8 (± 1.8)	7.0 (± 2.0)	*p* = 0.005

Presented as mean (± SD). s’, peak systolic velocity; e’, peak early diastolic velocity; a’, peak late diastolic velocity.

**TABLE 3:** Individual age gap between biological and chronological age.

	Low-risk	High-risk, total	Hypertension	Diabetes	IHD	Cardiac event
	**(*n* = 881)**	**(*n* = 882)**	**(*n* = 742)**	**(*n* = 179)**	**(*n* = 231)**	**(*n* = 202)**
Systolic peak	0 (ref.)	15 (10–20) years	16 (10–21) years	11 (1–20) years	16 (8–25) years	21 (12–30) years
		*p* < 0.001	*p* < 0.001	*p* = 0.02	*p* < 0.001	*p* < 0.001
Early diastole	0 (ref.)	8 (6–10) years	9 (7–11) years	7 (4–10) years	8 (6–11) years	8 (5–11) years
		*p* < 0.001	*p* < 0.001	*p* < 0.001	*p* < 0.001	*p* < 0.001
Average	0 (ref.)	12 (8–15) years	12 (9–15) years	9 (3–14) years	12 (7–17) years	15 (10–20) years
		*p* < 0.001	*p* < 0.001	*p* = 0.002	*p* < 0.001	*p* < 0.001

Individual age gap calculated as biological age subtracted by chronological age. Computed based on the two areas of the TDI curve significantly associated with risk group and age (systolic peak and early diastole). Presented as mean age gap in years (95% confidence intervals). *P*-value computed in comparison to low-risk group based on same TDI area.

All data analyses were performed in R statistical software version 4.0.5 (15).

## Results

The study population consisted of 1,763 persons of which 42% (*n* = 742) had hypertension, 10% (*n* = 179) had diabetes, and 13% (*n* = 231) were known with IHD. During 10-year follow-up (IQR 9.8–10 years), 11.5% (*n* = 202) had a cardiac event defined as cardiac death or admission due to myocardial infarction or heart failure.

The study population was divided into two groups: A low-risk group (*n* = 881) without cardiac risk factors at baseline (hypertension, diabetes, and/or IHD) and without any cardiac events during a 10-year period, and a high-risk group comprising the rest (*n* = 882) (see Table 1).

### TDI curves in low-risk population

There was a significant association between age and peak TDI velocities in both risk groups, as presented in Table 2. In the low-risk population, automated whole-cycle analysis generated reference TDI velocities for any given age. In Figure 1, reference TDI curves are presented for ages of every 10 years.

As indicated by the background colors of Figure 1, there was a significant association between age and TDI-values throughout most of the heart cycle. Thus, Figure 1 illustrates how TDI curves change according to chronological age in low-risk individuals. Four phases of the heart cycle where the velocity trace differed most according to age were identified (*p* < 10^–10^; dark blue areas): Systolic peak (0.09–0.13 s), systolic plateau (0.18–0.27 s), early diastole (0.43–0.54 s) and late diastole (0.88–0.95 s). With increasing age, systolic velocities decreased, early diastolic velocities decreased and their peak was delayed, and late diastolic velocities increased until age 70 and then stopped increasing (absolute velocity values are considered when defining an increase/decrease here and in the following).

### TDI curves in high-risk population

**FIGURE 2:**
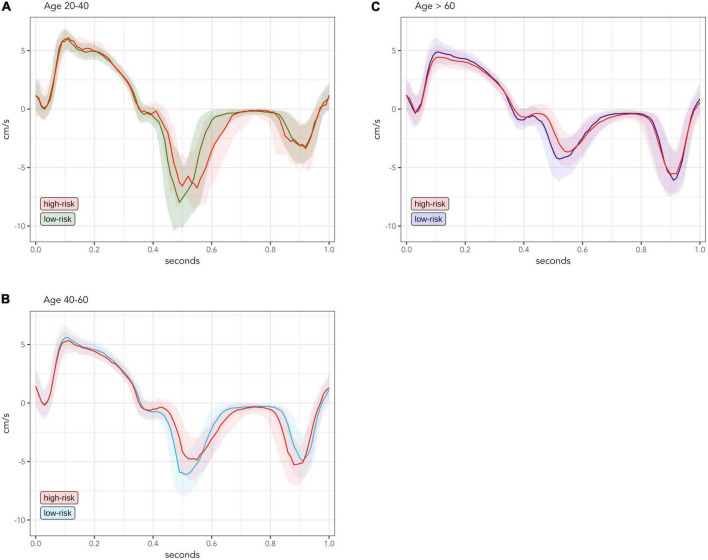
Comparison of median and interquartile range of measured TDI-values between low-risk and high-risk groups, divided into age groups of 20–40-year-olds [**(A)**; low-risk *n* = 226; high-risk *n* = 22], 40–60-year-olds [**(B)**; low-risk *n* = 432; high-risk *n* = 216] and > 60 year-olds [**(C)**; low-risk *n* = 223; high-risk *n* = 644]. TDI values are averaged for septal and lateral mitral sites, presented for one standardized heart cycle of 1 s. Solid lines are median values. Transparent colored areas represent interquartile range.

Figures 2A–C compare the median TDI curve for low-risk and high-risk populations within the same age groups. In the age group of 20–40-year-olds (Figure 2A), the high-risk population was very small (*n* = 22) and thus the signal of the TDI curve is less smooth. However, the high-risk group seems to have lower early diastolic velocity.

**FIGURE 3:**
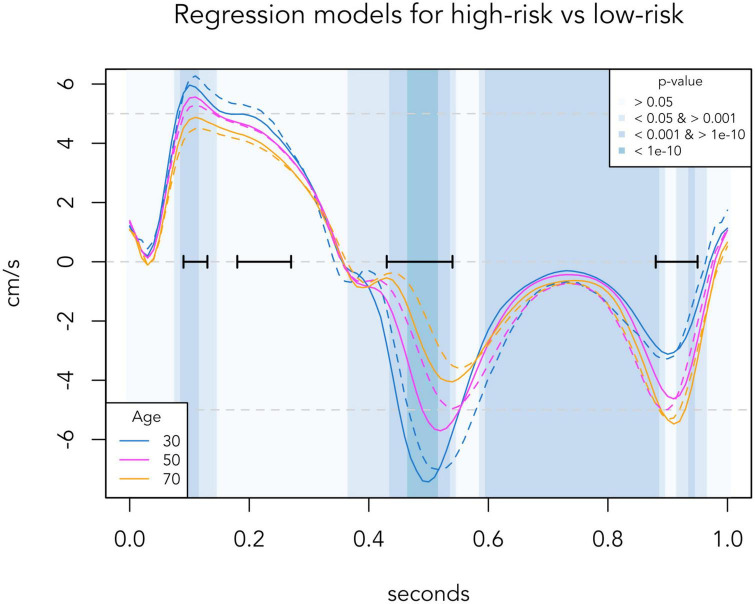
Regression-based TDI curves of selected age groups, comparing the low-risk population (solid lines) with the corresponding age group in the high-risk population (dashed lines). Background colors indicate significance levels of the age-adjusted association between risk group and TDI values at each 0.01 s time-position. Black horizontal bars indicate the four phases identified in Figure 1, where the TDI values differed most according to age. TDI values are averaged for septal and lateral mitral sites, presented for one standardized heart cycle of 1 s.

In the high-risk group of 40–60-year-olds (Figure 2B), early diastolic velocity is clearly lower and late diastolic velocity is slightly higher. Peak systolic velocity was also lower, while the systolic plateau remained the same compared to the low-risk group.

In the high-risk group older than 60 years (Figure 2C), the decrease of early diastolic and systolic velocities was further accentuated, now also involving the systolic plateau. However, the late diastolic velocity reached a turning point and now began to decrease compared to the low-risk group.

### Distinction between TDI curves in high-risk and low-risk populations

Figure 3 presents automated whole-cycle analysis of the data presented in Figure 2. The regression-based reference velocities of the low-risk group (as presented in Figure 1) were compared to the regression-based velocities of the high-risk group for the corresponding age at each time point. To ease visual comparison to Figure 2, TDI curves of 30-, 50-, and 70-year-olds are illustrated. For the entire population, of the four important phases identified in Figure 1, only the systolic peak (*p* = 0.002) and early diastole (*p* < 0.001) differed significantly between the low-risk and high-risk population, as velocities during these periods were lower in high-risk individuals.

### Biological age vs. chronological age

As a tool to discriminate high-risk individuals from low-risk individuals, we present a concept of assessing the individual age gap between the TDI-derived biological age and the real chronological age of each individual. For each individual, biological age was calculated based on their mean TDI values in the two important phases of the heart cycle significantly associated with risk group and age (systolic peak and early diastole), and the corresponding individual age gaps are presented in Table 3 for each cardiac risk group and for the high-risk group as a whole.

Example for reading Table 3: An individual with a chronological age of e.g., 60 years and known hypertension, has systolic peak velocities like a 76-year-old low-risk individual (real age + 16 years) and early diastolic velocities like a 69-year-old (real age + 9 years). On an average, this 60-year-old individual with hypertension has a heart in the same condition as a 72-year-old low-risk individual (real age + 12 years).

Table 3 shows that all cardiac risk populations had a higher biological age than their chronological age, when looking at the systolic as well as the early diastolic TDI values.

## Discussion

In this study, we present a novel and automated whole-cycle approach to analyzing TDI velocity curves and apply this analysis to a large group of individuals from the general population. In this way, four highly significant phases of the cardiac cycle that differ the most with age were identified: The systolic peak, the systolic plateau, early diastole, and late diastole. Using the same type of analysis, we demonstrate significant differences between low-risk and high-risk individuals during two of these phases, as TDI velocities of high-risk and low-risk individuals differ significantly at the systolic peak and early diastolic part of the TDI curve. We found that individuals with cardiac risk factors display findings compatible with an accelerated aging of the heart, and we propose a way of quantifying the individual age gap between the TDI-derived biological age and the chronological age.

### Myocardial velocities in healthy aging

In Figure 1, we show how whole-cycle TDI curves change in normal healthy aging of the heart. Using a regression model on standardized curves corrected for heart rate, we found that there was a significant association with age throughout most of the TDI curve, but with large differences in the strength of the association during different phases of the cardiac cycle. We identified four highly significant phases: The systolic peak, the systolic plateau, early diastole and late diastole. The general pattern of age-related changes—an early shift of diastolic velocities while systolic velocities and thus the total heart movement remain preserved until older age—is similar to previous findings (8, 9).

This presentation of standardized reference values for the continuous TDI curve, and the identification of highly significant areas, made it possible to compare the changes in healthy aging in low-risk individuals with the differences in myocardial velocities seen in high-risk individuals.

### Changes to myocardial velocities associated with cardiac risk factors

To evaluate the effects of cardiac risk factors separately from normal age-related changes, we compared whole-cycle TDI curves between low-risk and high-risk individuals within each age group (Figure 2). Even though our study is cross-sectional, the distinct TDI differences between low-risk and high-risk individuals in each age group could be seen as the chronological development of different TDI changes associated with duration of cardiac risk factors, since older individuals are likely to have longer-lasting contribution from risk-factors than younger individuals (16).

Our data suggest that the earliest degenerative change associated with cardiac risk factors is a decrease in early diastolic velocities. Later, there is an increase in late diastolic velocities and decrease in peak systolic velocities. With further duration, these changes are accentuated, and velocities of the systolic plateau also begin to decrease. However, the increase of late diastolic velocities will reach a turning point after which they will start to decrease, which makes the late diastolic phase less suited for discrimination between individuals at high and low risk.

These changes seen in Figure 2 are also well illustrated in the regression-based curves of Figure 3. Surprisingly, in the regression curve of the youngest group, the whole systole seems to be higher in the high-risk group than the low-risk group. However, this deviation of the regression model is likely due to the small number of high-risk individuals aged 20–40 years (*n* = 22) and should be interpreted with caution. From the regression model, we identified two important phases of the heart cycle that were both strongly associated with age and also significantly differed between risk groups after adjusting for age. Thus, our data confirm that the systolic peak and the early diastole are the best phases of the TDI curve to distinguish high-risk individuals from low-risk individuals, as previously suggested by other studies (11, 17, 18).

### Individuals at high risk of cardiac events show signs of accelerated aging of the heart

Held together, our whole-cycle analyses demonstrate that the changes in TDI velocities associated with cardiac risk factors in subjects within the same age group appeared to be similar to the changes of the TDI curve seen in healthy aging. Thus, it suggests that conditions associated with increased cardiac risk cause a general accelerated aging of the heart. In the literature, the idea of a disease-related acceleration of normal cardiac aging is also suggested in a study on hypertension (19).

Consequently, it might become possible to screen for high-risk patients by taking the TDI curve of any given patient and compare it to a set of normal reference TDI curves. If the patient’s TDI curve corresponds to an older age than expected, then the patient is likely to have some accelerated cardiac deterioration and thus be at an increased risk of cardiac event.

To illustrate this potential application, we built a model to estimate biological heart age based on the two highly significant areas of the reference TDI curves (systolic peak and early diastole). Based on this, we present a quantification of the individual age gaps between biological age and chronological age for different risk groups (Table 3). With further studies and software development it might be possible to use this approach to identify high-risk patients during echocardiography by comparing the measured TDI curve with built-in reference TDI curves.

Compared with 2D strain deformation imaging, TDI offers a number of potential advantages. TDI directly measures motion, and the temporal resolution is high, which allows a reproducible determination of peak motion during individual phases of the cardiac cycle. Only measurements from the mitral sites are needed for TDI, and the examination is thus feasible in most patients even when image quality is suboptimal.

### Study strengths

This study has certain strengths. 1) The unique extensive national Danish registries made it possible to have a 100% complete follow-up of our large study population of 1,763 individuals. 2) This study is the first study to analyze whole-cycle TDI velocity curves. Our method of correcting for heart rate made it possible to compare standardized whole-cycle curves for any individual in an automated manner and thus allowed the hypothesis-free identification of important phases of the heart cycle.

### Study limitations

This study has certain limitations. 1) The inhabitants of Denmark and thus the study population are predominantly Caucasian, which may limit the generalizability of our findings. 2) Standardizing velocity curves of different heart rates is a theoretical approach that might not be an exact reflection of reality, thus risking a slight time-shift of individual peak values, resulting in lower mean peak values in the standardized reference values. An alternative to our approach would be to identify temporal valve events and use these to define periods of the cardiac cycle, which would also better allow analysis of the isovolumetric phases. This approach would require manual analysis and thus be less suited for the kind of automated analysis we have developed here. 3) Averaging the septal and lateral wall velocity curve serves to reduce variability but might hide differences between the two walls.

## Conclusion

In the general population, TDI myocardial velocities are significantly associated with age throughout most of the cardiac cycle, and individuals with cardiac risk factors display TDI changes compatible with a general accelerated aging of the heart. The systolic peak and early diastolic part of the TDI curve are best at distinguishing between low-risk individuals and high-risk individuals and may thus be used to calculate a TDI-derived biological age—a potential tool for identifying high-risk patients at risk of future cardiac events.

## Data availability statement

The datasets presented in this article are not readily available because individual-level data are identifiable data and cannot be shared publicly. However, relevant intermediate-level data can be made available on request and following appropriate ethical review. Requests to access the datasets should be directed to corresponding author.

## Ethics statement

The studies involving human participants were reviewed and approved by the regional ethics committee. The patients/participants provided their written informed consent to participate in this study.

## Author contributions

All authors have contributed significantly to all parts of the submitted work (study concept and design, analysis and interpretation of data, and drafting and revision of the manuscript).
